# Validation and Comparison of BAP-65, DECAF, NEWS, and a Novel Combined Score for Predicting in-Hospital Mortality in Patients with Acute Exacerbation of COPD

**DOI:** 10.2147/COPD.S546523

**Published:** 2025-10-15

**Authors:** Maria Boesing, Giorgia Lüthi-Corridori, Laurin Manuel Sarbach, Fabienne Jaun, Daniel Olejar, Michael Brändle, Jörg D Leuppi

**Affiliations:** 1 University Institute of Internal Medicine, Kantonsspital Baselland, Liestal, Switzerland; 2 Faculty of Medicine, University of Basel, Basel, Switzerland; 3 Executive and Continuing Education, Harvard T. H. Chan School of Public Health, Boston, MA, USA; 4 Department of Internal Medicine, HOCH Health Ostschweiz, Kantonsspital Sankt Gallen, Sankt Gallen, Switzerland

**Keywords:** COPD, in-hospital death, risk stratification, score, AECOPD-COMBI

## Abstract

**Purpose:**

Acute exacerbations of chronic obstructive pulmonary disease (AECOPD) are a major cause of hospital admissions and are associated with significant morbidity and mortality. Several scoring systems are available for early risk stratification, such as DECAF, BAP-65, and NEWS, each incorporating parameters from different clinical domains. This study aimed to validate and compare established risk scores for predicting in-hospital mortality in patients with AECOPD and to assess the accuracy of a novel composite model.

**Patients and Methods:**

The BAP-65 score, a modified version of the DECAF score (DECAFm), and the National Early Warning Score (NEWS) were calculated using admission data of patients hospitalized for AECOPD in a Swiss hospital in 2022 and 2023. Predictive power for in-hospital death was compared using receiver operating characteristic (ROC) curves and the respective area under the curve (AUC). A novel scoring system, AECOPD-COMBI, combining parameters used in the three scores, was validated in the same cohort.

**Results:**

314 patients (mean age 73 years (range 48–94), 47% female) were included, of whom 7 died during hospitalization (2.2%). Patients who died had significantly higher scores at admission across all validated tools. Among the established scores, the BAP-65 performed best in the prediction of in-hospital death (AUC 0.79), followed by DECAFm (AUC 0.72) and NEWS (0.64). The novel AECOPD-COMBI reached the highest AUC of 0.9 and, when setting the high-risk score threshold to *S*=18.5, it demonstrated strong classification accuracy (sensitivity 100%, specificity 81%, accuracy 81%).

**Conclusion:**

The AECOPD-COMBI score showed promising potential in identifying patients at risk of in-hospital death, potentially outperforming established scores. While the cohort’s low event rate may have influenced predictive estimates and performance differences were not statistically significant, these findings still highlight the score’s potential value in clinical decision-making. Given the small sample size and preliminary nature of the study, these results should be interpreted with caution. Larger studies are needed to validate the score’s applicapbility and assess its performance for other relevant outcomes.

## Introduction

Chronic obstructive pulmonary disease (COPD) is the third leading cause of death globally.^1^ Acute exacerbations of COPD (AECOPD) are a major cause of emergency hospital admissions and are associated with substantial morbidity and healthcare costs.^1^,^2^ AECOPD is linked to an accelerated decline in lung function and is a strong predictor of subsequent exacerbations and COPD-related mortality.^2–4^ Hospitalized patients with AECOPD face in-hospital mortality rates ranging from 3.5% to 16%.^5–7^ While the Global Initiative for Chronic Obstructive Lung Disease (GOLD) publishes evidence-based up-to-date recommendations for the management of AECOPD annually,^1^ their implementation in clinical practice remains limited, especially regarding intensified treatment, such as non-invasive ventilation (NIV), in the more severe cases.^8^,^9^ Early risk stratification is crucial to inform clinical decision-making, guide appropriate allocation of resources, and potentially improve patient outcomes. An early risk stratification in patients hospitalized for AECOPD enables targeted management strategies, potentially improving outcomes by prioritizing intensified monitoring, prompt initiation of evidence-based treatment, or additional diagnostic evaluation for high-risk patients, whereas patients identified as low-risk can often be safely managed on general wards with early mobilization and discharge planning.

However, accurately identifying patients at greatest risk upon admission remains challenging. Several clinical factors have been shown to be associated with poor outcomes in patients hospitalized for AECOPD, including advanced age, cardiovascular comorbidities, altered mental status, and certain abnormal laboratory findings.^10–12^ Over the years, various risk stratification tools, such as the BAP-65 (elevated blood urea nitrogen, altered mental status, pulse >109, age ≥ 65) score,^12^ the DECAF (Dyspnea, Eosinopenia, Consolidation, Acidemia and atrial Fibrillation) score,^13^ and the Ottawa COPD Risk Scale^11^ have been specifically developed to predict adverse outcomes in AECOPD, including in-hospital mortality.^11–13^ Other non-specific scores, such as the National Early Warning Score (NEWS) and the CURB-65 (Confusion, Urea, Respiratory rate, Blood pressure, and age ≥ 65 years) score, have been shown to perform well in predicting adverse outcomes in AECOPD.^14–16^ However, each scoring system incorporates different clinical parameters, and their performance varies across populations and settings.^10–17^

Despite their demonstrated clinical utility, available risk scores also have certain limitations. Some were developed or validated over a decade ago, prior to the widespread use of newer evidence-based therapies and updated guidelines for the management of AECOPD, which may influence patient outcomes. In recent years, significant advances such as the broader use of NIV, updated protocols for the duration of corticosteroid use, more individualized antibiotic stewardship, and expanded access to long-acting bronchodilators and combinations of inhaled therapies have reshaped clinical practice.^1^,^18–20^ As a result, there is a lack of recent validation of risk scores in contemporary patient populations treated under current standards of care. Additionally, increased attention to comorbidity management, rehabilitation strategies, and early discharge planning may also affect prognosis.^21^,^22^ Moreover, risk stratification tools differ considerably in the parameters they incorporate – such as patient history and demographics, vital signs, instrumental diagnostics and laboratory findings – which contributes to variability in their performance across different clinical settings.^10^,^13^,^15^ To date, few attempts have been made to combine the most predictive elements from multiple well-established scores into a single model. Moreover, to our knowledge, such an approach has not yet been externally validated. Further efforts to refine and validate risk stratification tools in contemporary patient cohorts may therefore contribute to the improvement of patient-centered care in AECOPD.

### Objectives

This study aimed to externally validate widely used scoring systems for the prediction of in-hospital mortality in AECOPD in a contemporary Swiss cohort. A secondary aim was the evaluation of a novel prediction model – AECOPD-COMBI – that integrates parameters from three different established scoring tools.

## Material and Methods

### Study Design and Setting

This study was a retrospective, observational, single-center study. Adult patients, who were hospitalized due to AECOPD for at least one night at the Cantonal Hospital Baselland, Switzerland (*Kantonsspital Baselland*, KSBL), between January 2022 and December 2023 and who fulfilled the eligibility criteria (see section 2.2) were included in the study. The KSBL is a public teaching hospital, serving a population of approximately 300’000 residents and managing around 23’000 inpatient cases per year.^23^,^24^ This manuscript was prepared in accordance with the STROBE (Strengthening the Reporting of Observational Studies in Epidemiology) guidelines.

### Study Population

Adult patients (aged 18 years or older) who were hospitalized for AECOPD for at least one night at the KSBL between January 2022 and December 2023 were eligible for inclusion in this study. Patients who did not consent to the use of their clinical routine data for research purposes (general research consent) were excluded.

### Outcomes and Scores

The outcome of interest was all-cause in-hospital death. Table 1 provides an overview of the scoring systems assessed in this study. The BAP-65 score,^12^ NEWS,^25^ and a modified version of the DECAF score^13^ (DECAFm) were retrospectively calculated with parameters available at the time of admission. Specifically, relevant vital signs, symptoms and records of mental status were taken from the emergency department (ED) documentation (first documented in-house measurement). For laboratory values, if applicable, the first in-house result within 24 hours after admission was used for the score calculations. Information about relevant comorbidities and patient history was taken from documented patient anamnesis and documented diagnosis lists from previous hospitalizations. Due to unavailability of the extended Medical Research Council dyspnea scale (eMRCD), which is necessary for the calculation of the DECAF score, the score calculation was modified as follows (DECAFm): No points were given for being too dyspneic to leave the house on a good day within the previous three months. The formulas for calculation of the assessed tools can be found in the respective publications.^12^,^13^,^25^

**Table 1:** Assessed Risk Stratification Tools for the Prediction of AECOPD Outcome

Tool	Original Purpose	Parameters
BAP-65	Prediction of in-hospital mortality / need for mechanical ventilation in patients > 40 years old hospitalized for AECOPD	BUN, altered mental status, pulse, age
DECAF	Prediction of In-hospital mortality in patients ≥ 35 years old hospitalized AECOPD^a^	eMRCD, eosinopenia, consolidation in chest x-ray, acidemia, atrial fibrillation
NEWS	Early detection of clinical deterioration after ED admission, hospitalized patients	Respiratory rate, SpO_2_, O_2_ supplementation, temperature, systolic blood pressure, heart rate, AVPU scale

**Notes**: ^a^ not to be used in patients with comorbidity expected to limit survival < 12 months.

**Abbreviations**: BAP-65, (elevated BUN, altered mental status, pulse > 109 beats/min, age > 65 years)-Score for AECOPD;^12^ DECAF, (Dyspnea, Eosinopenia, Consolidation, Acidemia, atrial Fibrillation)-Score for AECOPD;^13^ NEWS, National Early Warning Score;^25^ AECOPD, acute exacerbation of chronic obstructive pulmonary disease; BUN, blood urea nitrogen; eMRCD, extended Medical Research Council dyspnea scale; ED, emergency department; SpO_2_, peripheral oxygen saturation; O_2_, oxygen; AVPU, alert – verbal – pain – unresponsive; CABG, coronary artery bypass graft; PCI, percutaneous coronary intervention.

To explore whether predictive performance could be improved beyond existing scoring systems, a combination score “AECOPD-COMBI” is introduced (Table 2), that integrates all individual parameters used across the three evaluated scores (BAP-65, DECAFm, and NEWS). This approach was based on the rationale that each score draws on distinct domains of clinical information, such as vital signs, laboratory values, mental status, and patient medical history. The combination of these heterogeneous parameters into a single score aimed to capture a more comprehensive representation of the patient’s clinical status upon admission and consequently to improve the prediction of the assessed outcomes. The construction of the new score was based on the following principles:

- The AECOPD-COMBI is a simple adapted sum of BAP-65, DECAFm, and NEWS scores.
- To ensure comparable weighting in the composite score, all contributing scores were min-max scaled such that their maximum possible contributions were approximately equal (16–18 points; 17 ± 1), thereby minimizing disproportionate influence from any single score.
- Parameters, that are used in more than one of contributing scores (heart rate and mental status), are only used once in the overall sum, giving priority to the more granular system.

**Table 2:** New AECOPD-COMBI Score for the Risk Stratification of Severe Course in Patients Hospitalized for AECOPD^a^

Parameter		Range	AECOPD-COMBI	Score Origin
Demographics	Age (years)	41 - 64	0	BAP-65
		≥ 65	6	
Vital Signs at admission	Heart rate (beats/minute)	≤ 40	3	NEWS
		41–50	1	
		51–90	0	
		91–110	1	
		111–130	2	
		≥ 131	3	
	SpO_2_ (%)	≤ 91	3	NEWS
		92–93	2	
		94–95	1	
		≥ 96	0	
	SpO_2_ measured with supplemental O_2_	No	0	NEWS
		Yes	2	
	Respiratory rate (breaths/minute)	≤ 8	3	NEWS
		9–11	1	
		12–20	0	
		21–24	2	
		≥ 25	3	
	Body temperature (°C)	≤ 35.0	3	NEWS
		35.1–36.0	1	
		36.1–38.0	0	
		38.1–39.0	1	
		≥ 39.1	2	
	Systolic blood pressure (mmHg)	≤ 90	3	NEWS
		91–100	2	
		101–110	1	
		111–219	0	
		≥ 220	3	
	Altered mental status	No	0	BAP-65
		Yes	6	
Laboratory and instrumental diagnostics at admission	BUN (mg/dL)	< 8.9	0	BAP-65
		≥ 8.9	6	
	Acidemia (pH < 7.3)	No	0	DECAFm
		Yes	4	
	Eosinopenia (eosinophils < 0.05 x 10^9^/L)	No	0	DECAFm
		Yes	4	
	Consolidation on chest X-ray	No	0	DECAFm
		Yes	4	
History	Atrial fibrillation (ECG at presentation or history)	No	0	DECAFm
		Yes	4	

**Notes**: ^a^ For score calculation, all points are summed up.

**Abbreviations**: BAP-65, (elevated BUN, altered mental status, pulse > 109 beats/min, age ≥ 65 years)-Score for AECOPD; DECAF, (Dyspnea, Eosinopenia, Consolidation, Acidemia, atrial Fibrillation)-Score for AECOPD; NEWS, National Early Warning Score; °C, degrees Celsius. mmHg, millimeters mercury; BUN, blood urea nitrogen; AECOPD, acute exacerbation of chronic obstructive pulmonary disease; SpO2, Peripheral oxygen saturation; O_2_, oxygen. ECG, electrocardiogram.

Table 2 presents the resulting formula of the AECOPD-COMBI score.

### Data Collection and Management

Patients hospitalized with a COPD-related diagnosis (ICD-10 category J44) for at least one night in 2022 or 2023 were identified from the hospital’s administrative database. The list was manually reviewed to confirm an AECOPD diagnosis. After verification of eligibility, patient outcomes and parameters for score calculations were extracted from the electronic health records. These included discharge reports, nursing documentation, emergency reports, ICU reports, and laboratory records. Data were managed in a REDCap® (Research Electronic Data Capture) database.

### Statistical Analysis

Patient data were analyzed descriptively and reported as absolute and relative frequencies for categorical variables, or as medians with interquartile range (IQR) for continuous variables. Risk scores were summarized using mean and standard deviation (SD) for the overall cohort and stratified by primary outcome. Group differences in scores were assessed using Student’s *t*-test.

For the score calculation, variables with missing values were imputed using the k-nearest neighbor algorithm.^26^ Predictive performance of the scores for outcomes was compared by means of receiver operating characteristic (ROC) curves and the respective area under the curve (AUC) with 95% confidence intervals (95% CI). The resulting ROC curves were compared pairwise with a Z-Test following DeLong’s method.^27^

Score thresholds for high-risk classification were defined at the border to the upper quartile of the cohort distribution (ie, the top 25% were classified as “high-risk”). In the established scores, which corresponded to previously published thresholds for the individual tools.^10^,^13^,^15^ Venn diagrams were used to illustrate the pairwise overlap between the classification of the established scores and the AECOPD-COMBI score at the defined high-risk thresholds. Additionally, a heat map was used to display the positive predictive values (PPV), negative predictive values (NPV), and classification metrics including sensitivity, specificity, and accuracy for each score at these thresholds.

All reported p-values are two-sided at a significance level of 0.05. Data imputation, analysis, and graphical presentation were performed with R version 4.1.0 using the packages “bnstruct”, “rms”, “pROC”, “ggplot2”, and “ggvenn”.^28^ As a sensitivity analysis, validations were additionally performed on the original, non-imputed data set.

## Results

### Patient Characteristics

Between January 2022 and December 2023, 389 patients were hospitalized due to AECOPD at the KSBL. After the exclusion of 75 patients who had denied the hospital’s general research consent, 314 patients were included in this study. All included patients were admitted via the hospital’s ED. Table 3 summarizes their characteristics. The median age was 74 years, ranging from 48 to 94 years, and 53.2% of the patients were male. The most prevalent clinical sign upon admission was increased dyspnea (n = 275, 87.6%), with nearly two-thirds of the patients (n = 188) being tachypneic (respiratory rate > 20 breaths per minute), and 139 patients (44.4%) requiring immediate oxygen supplementation upon presentation. Although 70.3% of patients exhibited elevated C-reactive protein levels suggestive of inflammation, only 11.1% presented with fever. Alveolar involvement, indicated by consolidation on the chest X-ray, was documented in 54 patients (17.9%). Cardiac involvement, suggested by signs of pulmonary congestion on the chest X-ray or acute ischemic signs on the electrocardiogram (ECG), was observed in 48 patients (15.4%).

**Table 3:** Patient Characteristics

		Overall (n = 314)	Missing (%)
**Demographics**	Age (years), median [IQR] / [range]	74 [67, 80] / [48, 94]	0
	Male sex, n (%)	167 (53.2)	0
**Symptoms at admission**	Increased cough, n (%)	125 (39.8)	0
	Increased dyspnea, n (%)	275 (87.6)	0
	Increased sputum volume, n (%)	29 (9.2)	0
	Change of sputum color, n (%)	42 (13.4)	0
**Vital signs at admission**	Heart rate (bpm), median [IQR]	90 [78, 103]	0.3
	SpO_2_ (%), median [IQR]	92.5 [88, 95]	0
	SpO_2_ measured with supplemental O_2,_ n (%)	139 (44.4)	0.3
	Respiratory rate (brpm), median [IQR].	22 [20, 26]	7.6
	Fever (body temperature ≥ 38°C)	35 (11.1)	6.7
	Systolic blood pressure (mmHg), median [IQR]	138 [122, 155]	1.3
	Altered mental status, n (%)	22 (7.1)	1.0
**Laboratory and instrumental diagnostics at admission**	Elevated C-reactive protein (> 5 mg/L), n (%)	215 (70.3)	2.5
	Elevated blood urea nitrogen (> 8.9 mmol/L), n (%)	8 (2.6)	3.2
	Acidemia (pH < 7.3), n (%)	29 (12.7)	27.1
	Eosinopenia (eosinophils < 0.05 x 10^9^/L), n (%)	102 (38.6)	15.9
	Consolidation on chest X-ray, n (%)	54 (17.9)	3.8
	Signs of pulmonary congestion on chest X-ray	43 (14.2)	3.8
	Acute ischemic signs on ECG	6 (2.1)	10.8
**History**	Current smoker, n (%)	143 (49.3)	7.6
	Pack years, median [IQR]	50 [40, 60]	14.3
	Charlson comorbidity index, median [IQR]	4 [3, 5]	0
	Atrial fibrillation, n (%)	65 (20.7)	0

**Abbreviations**: IQR, interquartile range; bpm, beats per minute; SpO_2_, peripheral oxygen saturation; O_2_, oxygen; brpm, breaths per minute; °C, degrees Celsius; mmHg, millimeters mercury; ECG, electrocardiogram.

Out of the 314 included patients, 7 (2.2%) died during their hospitalization, 17 (5.4%) died within 30 days after admission, and 54 (17.2%) were admitted to ICU. The median length of hospital stay of patients who were discharged alive was 6 days (IQR [4, 9]). Table 4 summarizes the scores of the overall population and of those who died during their hospitalization. Compared to the overall population, BAP-65, DECAFm, and NEWS scores were significantly increased among deceased patients. The AECOPD-COMBI scores were substantially higher among the deceased patients than in the overall population (22.3 ± 3.7 vs 14.9 ± 5.5, *p* = 0.001).

**Table 4:** Scores at Admission, Overall and by Primary Outcome, Mean ± Standard Deviation and Significance Level

Score	Overall	In-Hospital Death	*p*-Value	Missing
	n = 314	n = 7		n (%)
BAP-65	2.2 ± 0.6	3.0 ± 0.8	<0.001***	13 (4.1)
DECAFm	0.9 ± 0.8	2.0 ± 1.1	0.001**	119 (37.9)
NEWS	5.5 ± 2.4	7.3 ± 2.8	0.049*	37 (11.8)
AECOPD-COMBI	14.9 ± 5.5	22.3 ± 3.7	0.001**	139 (44.3)

**Notes**: * p < 0.05, ** p < 0.01, *** p < 0.001 (Student’s *t*-test comparing outcome groups to the overall population).

**Abbreviations**: BAP-65, (elevated BUN, altered mental status, pulse > 109 beats/min, age > 65 years)-Score for AECOPD; DECAFm, modified (Dyspnea, Eosinopenia, Consolidation, Acidemia, atrial Fibrillation)-Score for AECOPD; NEWS, National Early Warning Score; BUN, blood urea nitrogen; AECOPD, acute exacerbation of chronic obstructive pulmonary disease.

### Prediction of in-Hospital Death

**Figure 1:**
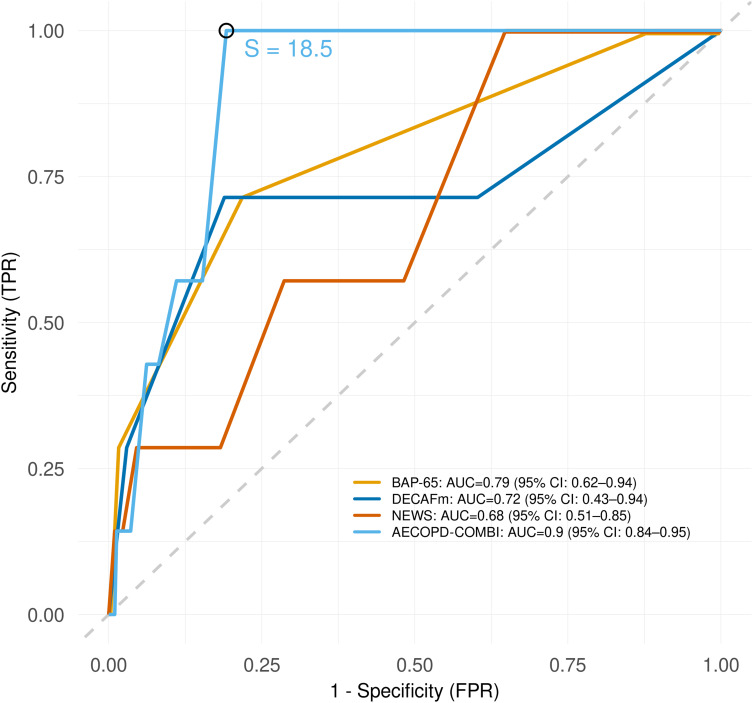
Receiver-operator characteristic (ROC) curves for the prediction of in-hospital death. AECOPD-COMBI threshold *S*=18.5 (3rd Quartile) is indicated for classification into high and low risk.

**Abbreviations**: BAP-65, (elevated BUN, altered mental status, pulse > 109 beats/min, age > 65 years)-Score for AECOPD; DECAFm, modified (Dyspnea, Eosinopenia, Consolidation, Acidemia, atrial Fibrillation)-Score for AECOPD; NEWS, National Early Warning Score. BUN, blood urea nitrogen; AECOPD, acute exacerbation of chronic obstructive pulmonary disease; AUC, area under curve; CI, confidence interval; TPR, true positive rate; FPR, false positive rate.

The predictive accuracy of the scores was assessed on the imputed dataset. Figure 1 presents the ROC curves of the analyzed scores for the prediction of in-hospital death. Among the established scores, BAP-65 had the highest accuracy with an AUC of 0.79 (95% CI: 0.62–0.94), followed by the DECAFm (AUC 0.72, 95% CI 0.43–0.94) and the NEWS (AUC 0.68, 95% CI: 0.51–0.85). Notably, at false positive rates above 65%, the NEWS showed higher sensitivity than the BAP-65 and the DECAFm (with NEWS < 5). The novel AECOPD-COMBI score showed better accuracy in predicting in-hospital death, with AUC of 0.90 (95% CI: 0.84–0.95), but the respective AUC differences were not statistically significant (BAP-65 vs AECOPD-COMBI *p* = 0.226). A true positive rate of 100% was reached at a score threshold of *S* = 18.5 and a comparably low false positive rate of 20%.

**Figure 2:**
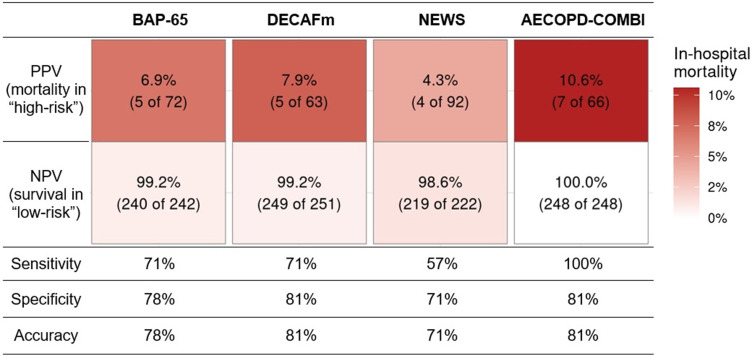
Summary of predictive values and classification accuracy for in-hospital death.

**Abbreviations**: BAP-65, (elevated BUN, altered mental status, pulse > 109 beats/min, age > 65 years)-Score for AECOPD; DECAFm, modified (Dyspnea, Eosinopenia, Consolidation, Acidemia, atrial Fibrillation)-Score for AECOPD; NEWS, National Early Warning Score; BUN, blood urea nitrogen; AECOPD, acute exacerbation of chronic obstructive pulmonary disease; PPV, Positive predictive value; NPV, negative predictive value.

Figure 2 summarizes the predictive values and classification accuracy at the pre-defined high-risk thresholds. The DECAFm score showed the highest overall accuracy (81%) for in-hospital death among the established scores, along with strong specificity (81%) and NPV (99%). The BAP-65 had slightly lower specificity (78%) and accuracy (78%), with the same sensitivity (71%). The NEWS showed the lowest overall accuracy (71%), sensitivity (57%), and PPV (4.3%) among all scores. The novel AECOPD-COMBI score demonstrated perfect sensitivity (100%) at the defined threshold of *S =* 18.5, with good specificity (81%) and accuracy (81%), and higher PPV (10.6%) than all of the established scores.

**Figure 3:**
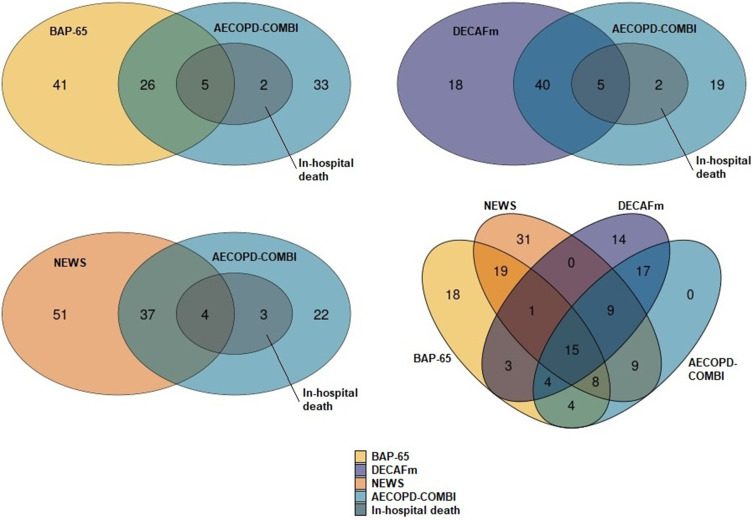
Overlap of high-risk classifications and actual in-hospital death (absolute numbers).

**Abbreviations**: BAP-65, (elevated BUN, altered mental status, pulse > 109 beats/min, age > 65 years)-Score for AECOPD; DECAFm, modified (Dyspnea, Eosinopenia, Consolidation, Acidemia, atrial Fibrillation)-Score for AECOPD; NEWS, National Early Warning Score; BUN, blood urea nitrogen; AECOPD, acute exacerbation of chronic obstructive pulmonary disease.

Figure 3 illustrates the overlaps between the high-risk classification of the scores and actual in-hospital death at the defined high-risk thresholds. While the AECOPD-COMBI classified all seven in-hospital death cases as “high-risk”, the BAP-65 and the DECAFm only classified five, and the NEWS only four of them as “high-risk”. The DECAFm had the biggest overlap with the AECOPD-COMBI with 45 patients being classified as “high-risk” by both scores. The respective overlap for BAP-65 and NEWS with the AECOPD-COMBI was 31 and 41 patients, respectively. All patients that were classified as “high-risk” by the AECOPD-COMBI were also identified as “high-risk” by at least one of the established scores.

## Discussion

This study evaluated the predictive performance of BAP-65, DECAFm, and NEWS in predicting in-hospital death of patients hospitalized for AECOPD in a recent Swiss population. Additionally, a newly developed composite score, AECOPD-COMBI, which combined the parameters of the three scores, was validated and its predictive performance was compared with that of the established scores. Our study has three main findings:

1. BAP-65, DECAFm, and NEWS showed moderate accuracy in predicting in-hospital mortality in our study population.
2. The novel AECOPD-COMBI score outperformed all three established scores in predicting in-hospital death.
3. At the score threshold *S* = 18.5, the AECOPD-COMBI achieved perfect sensitivity while still maintaining good specificity and capturing all patients classified as “high-risk” by any of the other three scores.

The characteristics of the 314 included AECOPD patients are consistent with previous studies. The median age of 74 years, sex distribution and clinical presentation reflects the typical population hospitalized for AECOPD.^6^,^12^,^13^,^29^ The observed in-hospital mortality (2.2%) and median hospital stay (6 days) align with previously reported ranges in similar cohorts.^6^,^10^,^12^ Overall, the study cohort seems to present a representative sample of patients hospitalized for AECOPD.

### Prediction of in-Hospital Death

In this cohort, seven patients (2.2%) died during hospitalization. The established scores (BAP-65, DECAFm, NEWS) were modestly elevated among these patients, consistent with their known value in in-hospital mortality prediction.^10^,^13^,^16^,^17^ Notably, the AECOPD-COMBI score was substantially higher within the group of deceased patients, suggesting its strong discriminatory ability.

Among the established tools, BAP-65 showed the best predictive performance (AUC 0.79), comparable to prior reports (AUC 0.76–0.79)^10^,^12^ The DECAFm performed worse in our cohort (AUC 0.72) than the original DECAF in previous validation studies (AUC 0.72–0.86), possibly reflecting the omission of the eMRCD.^13^,^16^,^17^,^30^,^31^ NEWS showed only moderate accuracy (AUC 0.68), in line with previous observations that it performs less well in disease-specific contexts.^15^

The novel AECOPD-COMBI score reached the highest predictive performance among all evaluated tools, with an AUC of 0.90 (95% CI: 0.84–0.95), suggesting both robust discriminative ability and potential clinical utility. Although differences in AUC between the AECOPD-COMBI and the established scores were not statistically significant, the higher point estimate with the narrowest CI supports the hypothesis of superior predictive power. In contrast, the wide CIs observed for the established scores likely reflect a limitation of the relatively small cohort. Given the relatively small sample size and low number of deaths, these findings must be considered preliminary and interpreted with caution, highlighting the need for larger studies.

At the predefined thresholds, the DECAFm score achieved the highest overall accuracy (81%) among the established tools and excellent NPV (99%), consistent with its reported utility in ruling out low-risk patients.^13^,^30^ The BAP-65 performed comparably (78% accuracy, 71% sensitivity), while NEWS showed the weakest performance (71% accuracy, 57% sensitivity, PPV 4.3%), underscoring its previously reported limitations when applied to disease-specific outcomes.^15^ In contrast, the AECOPD-COMBI achieved perfect sensitivity (100%) at the high-risk threshold *S* = 18.5, good specificity (81%) and superior PPV (10.6%). It correctly identified all deaths (n = 7) as “high-risk”, whereas BAP-65 and DECAFm each missed two cases and NEWS missed three.

The overlap analysis showed varying degrees of agreement between the established scores and the AECOPD-COMBI, reflecting differences in the clinical parameters prioritized by each tool. Importantly, the AECOPD-COMBI did not flag any additional patients as “high-risk” beyond those already recognized by the established tools. This suggests its potential for the reliable identification of all patients at risk of in-hospital death while minimizing false positives – a key advantage in clinical risk stratification, especially in high-stakes settings like the ED.

Its multidimensional structure acknowledges the multifactorial nature of mortality risk in AECOPD and may explain the AECOPD-COMBI’s superior performance. In the context of digitalized healthcare systems, automatic parameter extraction from electronic health records can facilitate its integration into clinical workflows.

## Limitations

This study has several limitations that should be considered when interpreting the results. First, the relatively small sample size, the single-center design, and in particular the low number of in-hospital deaths, limits the statistical power and generalizability of the findings. This constraint likely contributed to the wide confidence intervals observed for the AUCs of the established scores and reduces the reliability of direct comparison between them. A direct comparison in a larger, multicenter population could potentially lead to more reliable estimators and reveal statistically significant differences in predictive performance. Additionally, a larger sample size would allow for a more granular identification of the main drivers of in-hospital mortality among the score’s parameters.

Second, the retrospective design introduces an inherent risk of missing or incomplete data. In particular, we were unable to calculate the original DECAF score due to the absence of the eMRCD documentation and therefore relied on a modified version (DECAFm), which may differ in prognostic accuracy and affect comparability. Missing values, despite being imputed, might have introduced some bias. However, sensitivity analyses of the non-imputed dataset yielded consistent findings. In addition to its impact on research validity, missing data also poses practical challenges for implementing a potential risk stratification tool in clinical routine. While certain parameters, like eosinophil count, can be readily obtained in most clinical settings, others, such as arterial blood gas analysis, may not always be available in the ED. This discrepancy may limit the tool’s feasibility in some practice settings and underscores the need for flexible tools that can offer meaningful guidance even when some inputs are unavailable.

Third, while the AECOPD-COMBI score demonstrated promising performance, it may benefit from further refinement. For example, incorporating the eMRCD score, an important marker of baseline functional status, could enhance its discriminatory ability. Additionally, the score does not currently include broader measures of patient vulnerability, such as the Charlson Comorbidity Index or previous severe exacerbations, which have been shown to influence outcomes in patients with COPD and other chronic diseases.^32^ Moreover, the tool’s performance may improve if binary parameters are redefined as ordinal variables in cases where a graded association with outcome is plausible. This applies particularly to parameters such as age, altered mental status, and acidemia. Such refinement could enhance the model’s ability to capture the risk for poor outcome more accurately.

Finally, this study focused solely on in-hospital mortality as an outcome. Other clinically relevant endpoints, such as the need for mechanical ventilation, ICU admission, or length of hospital stay were not included in the analysis but may provide important complementary insights into the utility of prognostic scores.

## Conclusion

The observed trends suggest that the multidimensional AECOPD-COMBI score may offer comparable or improved clinical utility over established tools in identifying patients at risk of in-hospital death. While this complexity might previously have had limited usability, the increasing digitalization of healthcare now allows for seamless extraction of parameters from electronic health records, facilitating real-time and automated score calculation. This makes the AECOPD-COMBI both comprehensive and practical, with the potential to support digitally integrated risk stratification in patients hospitalized for AECOPD. The score’s high sensitivity and specificity, robust performance and overlap with validated tools such as the BAP-65, DECAF, and NEWS support its potential integration into clinical decision-making. However, the limited number of in-hospital deaths in the current validation cohort, and the resulting low statistical power restrict the strength of these preliminary conclusions. Larger studies in independent, multicenter cohorts are needed to confirm the generated hypotheses, further refine the AECOPD-COMBI, define robust cutoff values, and assess its predictive performance across different settings before clinical adoption. Future research should also explore its utility in predicting additional clinically relevant outcomes, such as mechanical ventilation and length of hospital stay.

## Data Availability

The data presented in this study are available on reasonable request from the corresponding author. The data are not publicly available due to restrictions in data privacy.
